# Supplementary material for: Biological Sensing of Nitric Oxide in Macrophages and Atherosclerosis Using a Ruthenium-Based Sensor
Source: Biomedicines. 2022 Jul 27;10(8):1807. doi: 10.3390/biomedicines10081807 (PMC9405170; doi:10.3390/biomedicines10081807)
Supplement: Supplementary file 1 [file biomedicines-10-01807-s001.zip › biomedicines-1762329-supplementary.pdf]

Supplementary materials

Biological sensing of nitric oxide in macrophages and atherosclerosis using a Ruthenium-based sensor

Achini K Vidanapathirana[1,2,4*], Jarrad M Goyne[1,2,4*], Anna Williamson[1,4], Benjamin J Pullen[1,2,4], Pich Chhay[1,4], Lauren Sandeman[1], Julien Bensalem[5], Timothy J Sargeant[5], Randall Grose[6], Mark J Crabtree[7], Run Zhang[3], Stephen J Nicholls[2,8], Peter J Psaltis[1,2,4,9] and Christina A Bursill[1,2,4†]

*Equal author contributions

Short Title: Nitric oxide sensor application in atherosclerosis

[1]Vascular Research Centre, Lifelong Health Theme, South Australian Health and Medical Research Institute, Adelaide, South Australia 5000, Australia

[2]Australian Research Council (ARC) Centre of Excellence for Nanoscale BioPhotonics (CNBP)

[3]Australian Institute for Bioengineering and Nanotechnology (AIBN), the University of Queensland, St Lucia, Queensland 4072, Australia

[4]School of Medicine, University of Adelaide, Adelaide, South Australia 5000, Australia.

[5]Lysosomal Health in Ageing, Hopwood Centre for Neurobiology, Lifelong Health Theme, South Australian Health and Medical Research Institute, Adelaide, South Australia 5000, Australia

[6]Cancer Program, Precision Medicine Theme, South Australian Health and Medical Research Institute, Adelaide, South Australia 5000, Australia

[7]Division of Cardiovascular Medicine, British Heart Foundation Centre of Research Excellence, Radcliffe Department of Medicine, University of Oxford, United Kingdom

[8]MonashHeart, Monash University, Clayton, Victoria 3168, Australia.

[9] Department of Cardiology, Central Adelaide Local Health Network, Adelaide, Australia

Supplementary Figures

Supplementary Figure S1

Unedited gels from Western blot assessments presented in Figure 2C

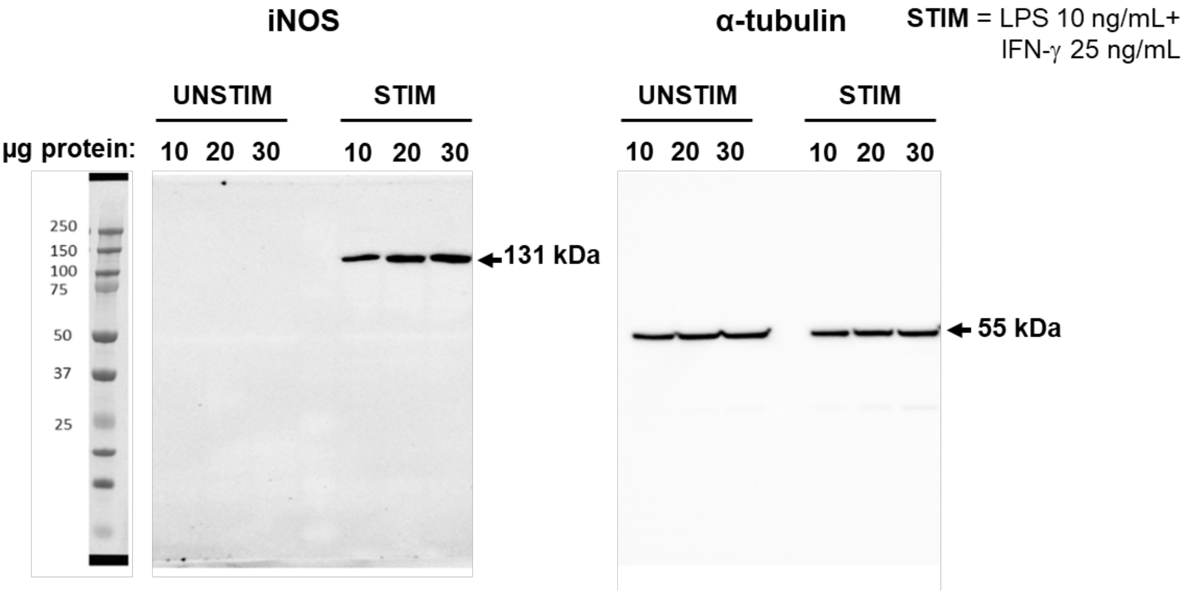

Supplementary Figure S2

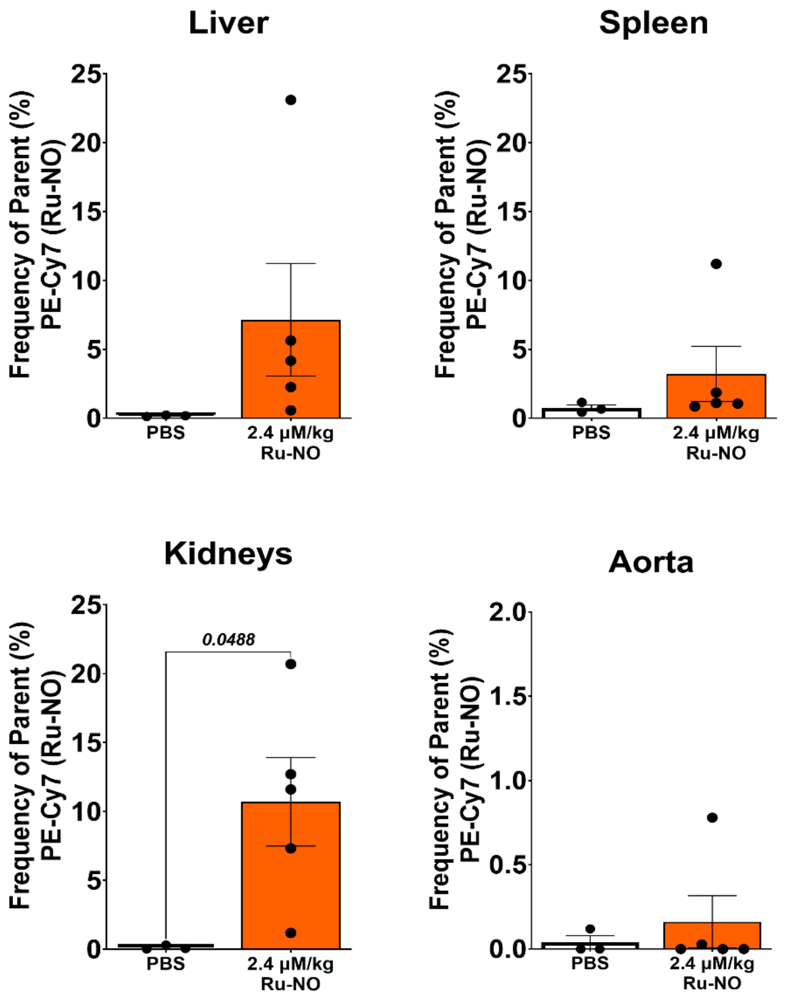

Supplementary Figure S2. **Biodistribution of Ru-NO *in vivo* following intravenous injection.** Ru-NO Fluorescence measured by flow cytometry in the liver, spleen, kidneys and aorta 24 h following intravenous administration of 2.4µM/kg Ru-NO to C57BL/6J mice. Mean ± SEM, *p* values were derived from an unpaired *t test (*n=3-5 mice/group)

Supplementary Figure S3

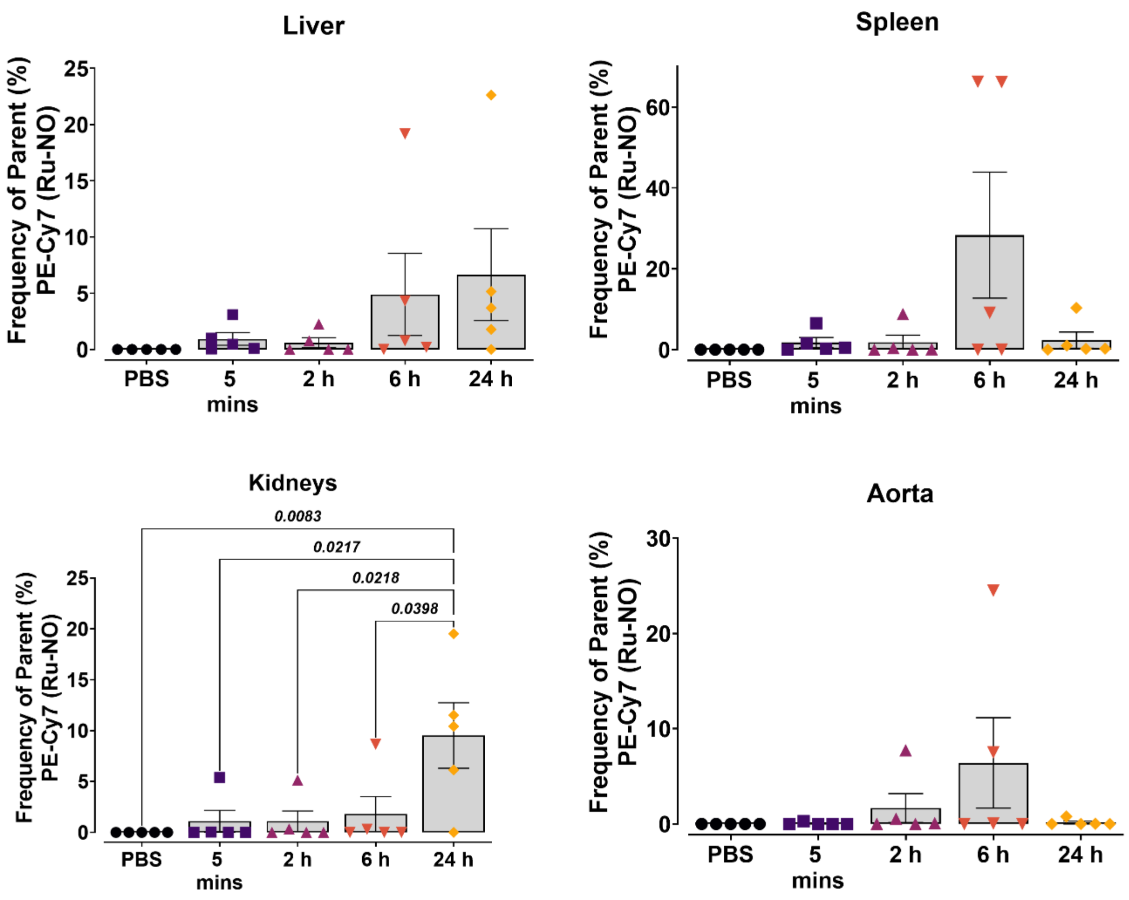

Supplementary Figure S3. Time course of Ru-NO fluorescence *in vivo* after intravenous infusion. Ru-NO fluorescence in the liver, spleen, kidneys and aorta at different timepoints following the intravenous administration of 2.4µM/kg Ru-NO to C57BL/6J mice. Mean ± SEM, *p* values were derived from a one-way ANOVA with a Tukey's post-hoc test (n=5 mice/group).

Supplementary Figure S4

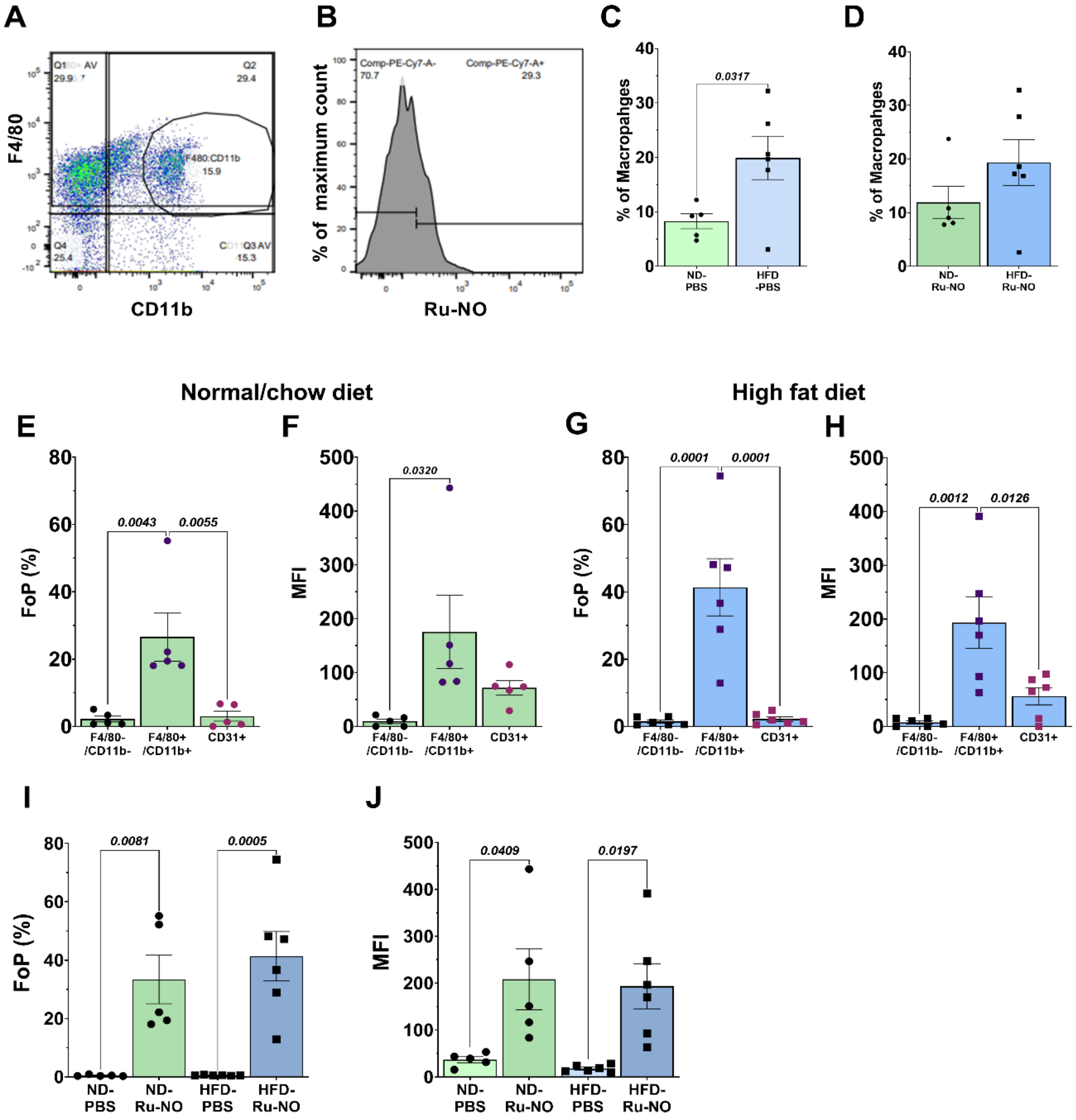

Supplementary Figure S4. ***Ex vivo*** **uptake of Ru-NO by macrophages in liver cell suspensions from atherosclerotic mice. A**. Plot of the distributions of liver cell suspensions incubated with antibodies against CD11b and F4/80 markers. **B.** Histogram demonstrating the shift in Ru-NO fluorescence in liver cell suspensions. Proportion of macrophages in all viable cells following **C.** PBS and **D.** Ru-NO. **E.** FoP and **F.** MFI for Ru-NO fluorescence in myeloid cells (CD11b^{+}F4/80^{-}), macrophages (CD11b^{+}F4/80^{+}) and endothelial cells (CD31^{+}) in chow fed mouse livers. **G.** FoP and **H**. MFI for Ru-NO fluorescence in HCD fed livers. **I.** FoP and **J**. MFI in CD11b^{+}F4/80^{+} macrophages comparing liver cell suspensions from chow and HCD-fed mice following PBS or Ru-NO. Mean ± SEM, *p* values from an unpaired t test or one-way ANOVA, with Tukey post-hoc test for multiple comparisons (n=5-6 mice/group).

Supplementary Figure S5

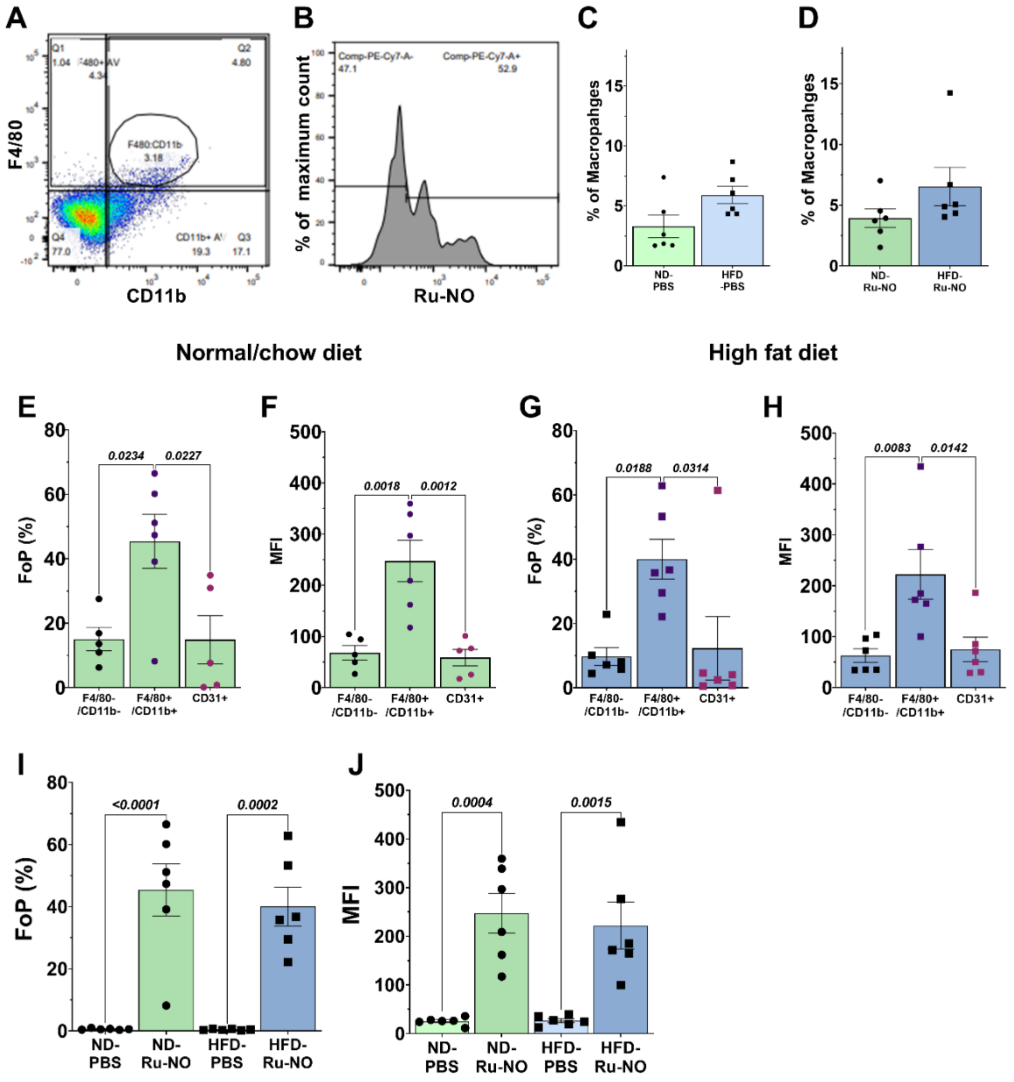

Supplementary Figure S5. ***Ex vivo*** **uptake of Ru-NO by macrophages in spleen cell suspensions from atherosclerotic mice. A**. Plot of the distributions of spleen cell suspensions incubated with antibodies against CD11b and F4/80 markers. **B.** Histogram demonstrating the shift in Ru-NO fluorescence in spleen cell suspensions. Proportion of macrophages in all viable cells following **C.** PBS and **D.** Ru-NO. **E.** FoP and **F.** MFI for Ru-NO fluorescence in myeloid cells ($CD11b^{+}F4/80^{-}$), macrophages ($CD11b^{+}F4/80^{+}$) and endothelial cells ($CD31^{+}$) in chow fed mouse spleens. **G.** FoP and **H**. MFI for Ru-NO fluorescence in HCD fed spleens. **I.** FoP and **J**. MFI in $CD11b^{+}F4/80^{+}$ macrophages comparing spleen cell suspensions from chow and HCD-fed mice following PBS or Ru-NO. Mean ± SEM, *p* values from an unpaired t test or one-way ANOVA, with Tukey post-hoc test for multiple comparisons (n=5-6 mice/group).

Supplementary Table.S1: Demographic and Clinical information of patient blood samples for the analyses with the Ru-NO sensor

Variables	Overall N = 50 (%)	No CAD n=19 (%)	Stable CAD n=20 (%)	MI n=11 (%)
**Gender**				
Male	35 (70.00)	12 (63.16)	17 (86.84)	6 (54.54)
Female	15 (30.00)	7 (36.84)	3 (19.34)	5 (45.45)
**Mean age (years) ± SD**	67.82 ± 11.17	64.05 ± 13.11	70.85 ± 7.407	68.82 ± 12.29
**Mean body weight (kg) ± SD**	85.12 ± 17.67	87.58 ± 19.66	86.35 ± 18.75	78.00 ± 8.79
**Mean BMI (kg/m^2) ± SD**	28.91 ± 5.53	28.75 ± 4.88	29.90 ± 6.82	26.86 ± 2.16
**Medical History**				
Diabetes mellitus	12 (24.00)	3 (15.78)	6 (30.00)	3 (27.27)
Hyperlipidaemia	29 (58.00)	10 (52.63)	13 (65.00)	6 (54.54)
Hypertension	27 (54.00)	7 (36.84)	12 (60.00)	8 (72.72)
Arrythmia/atrial fibrillation	9 (18.00)	3 (15.78)	5 (25.00)	1 (9.09)
MI (prior admission)	13 (26.00)	0 (0.00)	10 (50.00)	3 (27.27)
Stroke (prior admission)	3 (6.00)	2 (10.52)	1 (5.00)	0 (0.00)
**Medications**				
Statins	33 (66.00)	10 (52.63)	18 (90.00)	5 (45.45)
ACE inhibitors/ARB	23 (46.00)	8 (42.10)	13 (65.00)	2 (18.18)
Beta blockers	17 (34.00)	7 (36.84)	9 (45.00)	1 (9.09)
Aspirin	24 (48.00)	7 (36.84)	12 (60.00)	5 (45.45)
Metformin	8 (16.00)	2 (10.52)	5 (25.00)	1 (9.09)
Nitrates	4 (8.00)	0 (0.00)	3 (15.00)	1 (9.09)
**Social History**				
Alcohol drinkers	16 (32.00)	7 (36.84)	7 (35.00)	2 (18.18)
Current smoker	8 (16.00)	2 (10.52)	2 (10.00)	4 (36.36)
Former smoker	25 (50.00)	7 (36.84)	15 (75.00)	3 (27.27)

ACE: Angiotensin Converting Enzyme, ARB: Angiotensin receptor blockers, BMI: Body Mass Index, CAD: Coronary artery disease, MI: Myocardial Infarction, SD: Standard Deviation

Supplementary methods

S1. Flow cytometry

Flow cytometry was used to detect *in vitro, ex vivo* and *in vivo* applications of the sensor. Uptake and detection capacity of the sensor was first demonstrated by flow cytometry in mouse and human macrophages under different *in vitro* stimuli. At the end of the exposure period, the monocytes/myeloid cells/macrophages were detached using 0.5% Trypsin ETDA (Life Technologies). In the *in vivo* studies, the tissues from each organ were digested using liberase as described above and the cells were re-suspended in flow cytometry staining buffer (BD Biosciences). The cells were washed in media with FBS and were co-stained with antibodies against F4/80 (FITC), CD11b (PE), CD86 (BV421) and CD206 (AF647) antibodies to identify the myeloid cell populations. An antibody against CD31 (APC) was used as a marker for endothelial cells in *ex vivo* studies. Antibodies were diluted in staining buffer and incubated for 30 min at 4°C in the dark. After incubation the cells were washed and resuspended in FACS fix (BD biosciences). A nuclear stain, DAPI (Sigma-Aldrich) was added 1:500, vortexed and left for 5 minutes before analyses with the BD FACSCanto II™ flow cytometer analyser (BD biosciences). Ru-NO has been shown previously to have a maximum excitation and emission spectrum of 450 nm and 615 nm, respectively[1, 2] . Ru-NO was excited by the 488 nm blue Laser and the emitted signal detected using the 780/60 bandpass filter. The data analysis was performed with Flowjo 10.7.1 software (BD biosciences).

S2. Confocal microscopy

Confocal microscopy was used in both *in vitro* and *in vivo* studies of the sensor. Monocytes/ macrophages were grown on glass bottom plates or on low binding plates (Corning) and were incubated with 50 μM Ru-NO for 24 h 37˚C in an incubator or live imaging chamber attached to microscope set-up. A nucleic acid stain, DAPI with mounting media (Vectashield, Abacus VEH1200) was added to demarcate the nucleus. In the *in vivo* studies the tissue sections were

fixed in OCT and sectioned at 5 μm using a CryoStat (Leica) and co-stained with DAPI. A Confocal Microscope (Leica TCS SP8X/MP) at λ_{ex}=473 nm and λ_{em}=565-645 nm was used for imaging which were analysed using LAS-X imaging software (Leica Microsystems Pty Ltd, NSW, Australia). Fluorescence intensity of different channels were quantified for each image using cell-profiler software.

S3. Mass Cytometry (cytometry by time of flight, CyTOF) analysis

THP-1 monocytes and macrophages were treated with 10 and 50 μM of Ru-NO and incubated for 24 h. At the end of treatment, the cells were trypsinised, resuspended in PBS and centrifuged at 300 x *g* in 4°C for 5 mins. The cell suspension was then incubated with 1:200 dilution of 10 μM Cell-ID Cisplatin 195Pt (Fluidigm) for 5 minutes. Cells were quenched by adding 10 ml Maxpar® Cell Staining Buffer (Fluidigm). Samples were centrifuged at 300 x *g* in 4°C for 5 mins. The cells were re-suspended in Maxpar® Cell Staining Buffer then stained with an antibody cocktail containing anti-CD45-154Sm, anti-CD3-170Er, anti-CD20-147Sm, anti-CD4-174Yb, anti-CD8-168Er, anti-CD14-160Gd and anti-CD16-165Ho (Maxpar® Human Peripheral Blood Basic I Phenotyping Panel Kit, 7 Markers). After incubation for 30 mins, the cells were centrifuged and washed in Maxpar® Cell Staining Buffer. The cells were fixed in fresh 1.6% paraformaldehyde in Maxpar® PBS (1:9) and incubated for a further 20 mins. The cells were centrifuged at 800 x *g* for 5 mins, the resulting pellet was resuspended in cell-intercalation solution (1:999 dilution of 125 μM Intercalator-Ir in Maxpar Fix and Perm Buffer) and incubated for at least 1 h at room temperature. Cells were washed and resuspended in Maxpar® Cell Acquisition Solution containing 0.1x EQ™ Four element Calibration Beads. Cell suspensions were analysed using the Helios CyTOF System (Fluidigm) and analysed using the CyTOF v7.0 and Flowjo software.

S4. Western Blot Analysis for iNOS

BMDMs were analysed for iNOS protein expression using Western Blot as previously described[3] using iNOS antibody (Abcam, ab49999) with α-tubulin (Abcam, ab40742) loading control. Briefly, 10-30 μg of protein was loaded onto a 4% to 12% gel (Invitrogen) and run at 120 mA for 1 h. The gels were transferred onto nitrocellulose and blocked for 1 hour in 10% non-fat milk powder. Anti-iNOS antibody was added at 1:1000 and incubated overnight at 4˚C. Blots were washed and the secondary antibody added for 1 h at room temperature before the blot was visualised using a ChemiDoc (BIO-RAD).

S5. Toxicity and distribution studies in mice

A pilot study was conducted to determine the optimum concentration of the Ru-NO for the *in vivo* studies, to understand the sensor distribution and to assess toxicity. C57BL/6J mice were fed on a chow diet, water provided *ad libitum* and were used for distribution studies at 4-12 weeks of age. Either 0.6 or 2.4 μM/kg of Ru-NO or vehicle control (PBS) was injected by tail vein and the mice were monitored for 24 h post-exposure. Mice were deeply anaesthetised using a single administration of 5% isoflurane via inhalation and whole blood collected via cardiac puncture. Organs and tissues were harvested immediately after exsanguination (aorta, heart, bone marrow, spleen, liver and kidneys). For *in vivo* Ru-NO detection, tissues were harvested and kept on ice in Iscove's Modified Dulbecco's Medium (IMDM) until processing. Each tissue was cut into multiple sections and added to pre-warmed Hanks' Balanced Salt solution with Liberase (1:100 dilution) and incubated for 45 mins at 37°C in 5% CO_2 for tissue digestion. Cold IMDM (Sigma Aldrich) with 10% FBS was added at the end of the digestion process, cells strained using a Greiner 40μm Easy strainer (Greiner) and centrifuged at 800 x *g* rpm for 5min. The cell pellet was resuspended in 1 mL of IMDM and then washed for staining and flow cytometry analysis to assess the presence of NO bound sensor. At the end of the pilot study a 2.4 μM/kg (40 μM) concentration was selected for *in vivo* studies. This selected concentration was then injected intravenously and the mice were humanely

killed at 5 min, 2, 4, 6 and 24 h after the injection to determine the time-course distribution of the sensor.

References

1. Vidanapathirana AK, Pullen BJ, Zhang R, Duong M, Goyne JM, Zhang X, Bonder CS, Abell AD, Bursill CA, Nicholls SJ, Psaltis PJ. A Novel Ruthenium-based Molecular Sensor to Detect Endothelial Nitric Oxide. *Sci Rep* 2019;**9**:1720.
2. Zhang R, Ye Z, Wang G, Zhang W, Yuan J. Development of a ruthenium(II) complex based luminescent probe for imaging nitric oxide production in living cells. *Chemistry* 2010;**16**:6884-6891.
3. Bailey JD, Shaw A, McNeill E, Nicol T, Diotallevi M, Chuaiphichai S, Patel J, Hale A, Channon KM, Crabtree MJ. Isolation and culture of murine bone marrow-derived macrophages for nitric oxide and redox biology. *Nitric Oxide* 2020;**100-101**:17-29.
